# Power Imbalances, Food Insecurity, and Children’s Rights in Canada

**DOI:** 10.3389/fpubh.2016.00117

**Published:** 2016-08-11

**Authors:** Alison Blay-Palmer

**Affiliations:** ^1^Centre for Sustainable Food Systems, Wilfrid Laurier University, Waterloo, ON, Canada

**Keywords:** rights, power, food insecurity, Canada, children, neoliberal

## Abstract

Increasingly, food is provided through an industrial food system that separates people from the source of their food and results in high rates of food insecurity, particularly for the most vulnerable in society. A lack of food is a symptom of a lack of power in a system that privileges free market principles over social justice and the protection of human rights. In Canada, the high rates of food insecurity among Canadian children is a reflection of their lack of power and the disregard of their human rights, despite the adoption of the United Nations (UN) Convention on the Rights of the Child in 1991 and ratification of the International Covenant on Social, Economic and Cultural Rights in 1976, which established the right to food for all Canadians. Dueling tensions between human rights and market forces underpin this unacceptable state of affairs in Canada. Gaventa’s “power cube” that describes different facets of power – including spaces, levels, and forms – is used to help understand the power imbalances that underlie this injustice. The analysis considers the impact of neoliberal free market principles on the realization of human rights, and the negative impacts this can have on health and well-being for the most vulnerable in society. Canadian case studies from both community organizations provide examples of how power can be shifted to achieve more inclusive, rights-based policy and action. Given increased global pressures toward more open trade markets and national austerity measures that hollow out social supports, Canada provides a cautionary tale for countries in the EU and the US, and for overall approaches to protect the most vulnerable in society.

The “neo-liberal era” – if that is the term we want – has been a time of looking away, a time of averting our gaze from the causes and effects of structural violence. Whatever term we use to describe our times, we cannot avoid looking at power and connections if we hope to understand, and thus prevent, human rights abuses. And when we look at and listen to those whose rights are being trampled, we see how political rights are intertwined with social and economic rights, or, rather, how the absence of social and economic power empties political rights of their substance … hiding this suffering, or denying its real origins, serves the interests of the powerful … The persistence of such suffering concerns all of us (1).

## Introduction

The industrial food system, driven by neoliberal market principles, has resulted in well-documented negative effects, including growing numbers of people who are food insecure and who suffer from escalating rates of diet-related disease, including diabetes and obesity (2–4). The relentless co-option by multinational interests of the economic benefits of the global food industry goes hand in hand with the abrogation of responsibility for the negative social and ecological externalities, including the direct/indirect contribution by agri-food to global greenhouse gases (GHG), declining farmer income security, and the increasing enclosure of the commons (5–7). The “Feed 9 billion” campaign is a case in point. The rationale of this campaign is that more technology will produce more food. In this commercially constructed scenario, projected population growth is being used to argue for increased technological intervention in agri-food through the application of genetically modified organisms (GMOs) as part of packaged seed and chemical interventions. Instead of assuming that more technology and increased production are the answers, a more relevant and telling question would be “How can the existing 7.3 billion people feed themselves” in a way that respects their rights to access healthy, culturally appropriate food (8). More technology to increase production does not need to be the first response as evidenced by the fact that, despite the current availability of high technology options and current production providing more than enough food for every person to eat a healthy diet, about 850 million people globally are food insecure and another 1 billion plus are obese or overweight (9, 10).

Worldwide, children are among the biggest victims of this food system failure. As a recent UNICEF report explains, “We cannot claim that children’s rights are being upheld when 17,000 children under the age of 5 die every day, largely from causes we know how to prevent” (11). Given these unacceptable realities, it makes sense to support a more sustainable food system (SFS) – one that is socially just, economically localized, ecologically regenerative, and enhances citizen engagement – as a pathway to a fairer, healthier, greener, prosperous future (12–15).

While some progress has been achieved in this direction, and we know more about *what* needs to happen, we are short on the *how* (16, 17). As an entry point into an analysis about the right to food as one dimension of SFS, the specific challenges considered in this paper include the dueling epistemologies of market-based versus rights-based approaches, how this facilitates/impedes the right to food for children, and interventions that could be put into place to help compel the State to meet its obligations. For, as Narula states:

… an essential problem with the human rights framework is that it necessarily relies on the willingness of the State to implement reforms. Such an approach assumes a self-executing, trickledown quality of the law wherein top-down processes can effectively navigate entrenched power dynamics (18) … the State and its ruling elite are not neutral agents of social change. To the contrary, State actors and domestic elites often stand to benefit from rights-violating policies and practices [see Ref. (7, 19)].

So, while we may want States to respect human rights, the reality is that they often do not. Given this reality, the related tensions explored in this paper include (1) the contradictory and oppositional ways food security is addressed at different times by different levels of government and civil society organizations (CSOs) and the hybridity that emerges from different opportunities and tensions and (2) the contingency of various actors and the degree of support they can offer to the realization of basic human rights, given the power they hold. We explore whether it is possible to shift power to CSOs so that more attention is paid increasingly to SFSs that improve access to food for children as one way to respect their rights. One conclusion of this paper is that there needs to be deliberate checks and balances, so that States are accountable, if they violate human rights within their borders, and that countries in the Global North are scrutinized and held to account. Coupled with existing international agencies, engaged CSOs could be stepping stones to empower individuals and communities and provide scrutiny of States. There are two closely intertwined considerations here. First is the *capacity* for CSOs to support and advocate for food security as part of realizing the rights of children. A related consideration is the *on-going viability* of alternative SFS initiatives. While not the total solution, CSOs that are empowered and have the capacity to act could be a key piece in achieving human rights in the face of State intransigence. By legitimizing and raising the profile of CSOs, they can contribute to framing the discourse, laws, policies, and programs related to human rights at multiple scales. Additionally, effectively networked CSOs could better solidify more robustly embedded rights-based principles as part of SFSs and help empower marginalized communities, so they can ensure their own food security and right to food. This demands a deliberate and strategically inclusive approach to open the door wider and enhance participation from, for example, children, women, aboriginal peoples, migrants, and workers.

Given the current prevalence of extreme neoliberal interests, the need for an approach that supports children’s rights and challenges neoliberal assumptions is clear. There are practical considerations that flow from this observation. For example, organizations that rely primarily on government funding are beholden to the government of the day and are likely ill positioned to take on a role critical of the government. And, as well-documented elsewhere, they are also victims of the persistent downloading of services without the needed dollars to carry out these programs. As a result, as Allen and Guthman discuss with respect to Farm to School programs in the United States, “… advocates are essentially producing neoliberal forms and practices *de novo*, most notably those associated with contingent labor relationships and private funding sources, and the spuriousness and unevenness that necessarily follows” [(20), p. 412]. However, if CSOs are able to persist despite shifts in government priorities, this could allow them the space to activate and help ensure accountability of governments and the improved realization of human rights in the longer term.

As discussed later in the paper, networked governance could provide the agency needed to give voice to marginalized communities as networks shift from passive structures to engaged actors (21), while joined-up policy offers a step toward more integration across multiple policy spheres (22). To this end, the paper unfolds in four sections. First, given the central theme of power to the analysis, Gaventa’s “Power Cube” is presented as a conceptual framework that offers points of reference for understanding power dynamics including the levels, spaces, and forms of power. The power cube provides a lens to identify existing centers of power and to find gaps where improvements to enhance a rights-based approach could be inserted. Next, we turn our attention to the specifics of the United Nations (UN) Convention on the Rights of the Child (CRC) with particular attention to the Canadian context. We, then, move to our third point, an overview of child-focused food initiatives in Canada that emerged from civil society, not-for-profit, and government organizations in the absence of attention from the federal government to respect, protect, and fulfill the rights of children. Questions are raised about the intersection of rights-based initiatives with the market-based approach of the State – in this case, community initiatives that help meet the needs of children and their families to access healthy food as a basic human right. Selected case studies demonstrate how marginalized communities can be included in the co-creation of robust, multifunctional food systems that rely on their communities of food to foster improved food security. These initiatives demonstrate how empowerment can be realized by moving beyond a closed use of power to one with more open access to decision-making and the inclusion of more voices from multiple institutions and scales. The paper ends with a review of power dynamics and a discussion of implications for work in Organization for Economic Cooperation and Development (OECD) countries as well as the Global South. First though, we turn our attention to Gaventa’s framing of power.

## The “Power Cube”

Gaventa and colleagues elaborated their “power cube” as a transformational tool to (1) enable a deeper analysis of how power facilitates and/or impedes change, (2) capture the dimensions and dynamics of the potential to change power dynamics, and then, (3) demonstrate how to facilitate change through enhanced citizen engagement (23). Given the challenge at hand, this offers a fertile ground for considering how to enhance the right to food through the recalibration of power relations. As Gaventa explains, the cube provides a framework to assess the potential for transformation so that practitioners and activists can consider and describe power structures and identify how to challenge them [(23), p. 25]. In this way, the power cube can be a tool for transformation.

**Figure 1:**
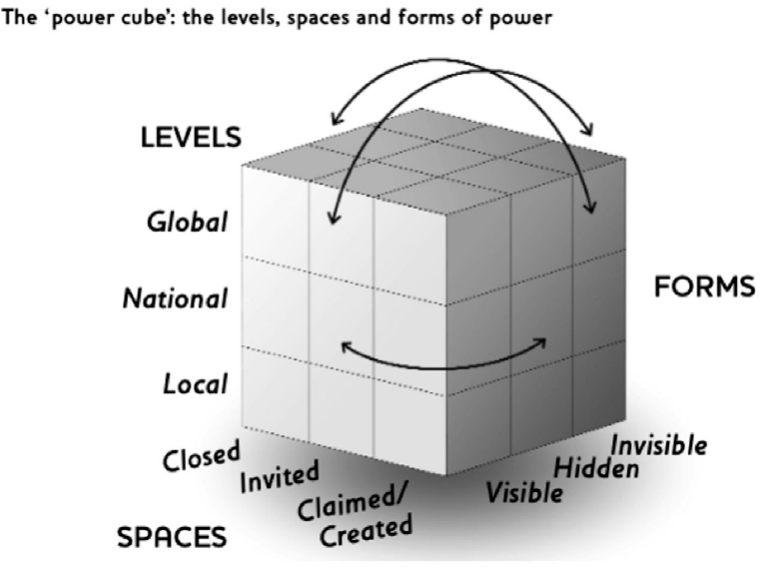
**Gaventa’s power cube (23)**.

The three-faceted cube includes the *spaces, levels*, and *forms* of power (Figure 1). *Spaces* of power include “opportunities, moments, and channels where citizens can act to potentially affect policies, discourses, decisions, and relationships that affect their lives and interests” [(23), p. 26]. Power spaces are socially constructed and facilitate and/or constrain one another (24). Using Cornwall’s work, Gaventa proposes a continuum ranging from “closed” through “invited” to “claimed/created” power spaces. This continuum moves from the most restricted, contained spaces where power is accessible exclusively to elite groups (social, economic, or political), to broader spaces that decision makers open or where they invite citizen or other actor participation, to spaces that are “claimed” by less powerful actors. Important considerations in the spaces of power are who occupies and controls the space and whether one space can be a platform to move into other spaces as a way to enhance power. Cautions about power and space include the potential for powerful actors to enclose and co-opt spaces of power from others, and the need for “staying power” by CSOs to capture and retain space.

The second face of the cube refers to *levels* of power: local, national, and international scales, as well as the micro-levels in between. Considerations here include the extent to which the level is appropriate and power is relevant at multiple and interacting levels so that, “the interrelationships of these levels of power with one another suggest that the challenge for action is not only how to build participatory action at differing levels, but how to promote the democratic and accountable *vertical links* across actors at each level” [(23), p. 28]. Questions of legitimacy and accountability that inform the extent of actual democratic engagement are also central to considering the interconnections across levels. The dynamic and contingent nature of power is particularly relevant for this paper as differences between levels can or could change and affect new dynamics at other levels. As Gaventa explains, “Many of these [levels] are shaped by the relevant legal frameworks of governmental administration, and may differ across … communities, yet increasingly, extra-local arenas seem to grow as centres of power and decision making” [(23), p. 28].

The third facet of the power cube is form. This captures the extent to which power is inclusive. Gaventa proposes three *forms* of power categories: visible, hidden, and invisible. Visible power includes the elements that reflect or support formal decision-making. This includes rules, laws, structures, and authorities. Hidden power refers to those people who are able to set the political agenda (25) and terms for the less powerful across multiple levels. Finding a place at this table is needed to influence what decisions are made. Finally, invisible power speaks to the shaping of culture and “… the psychological and ideological boundaries of participation … [that shape] people’s beliefs, sense of self and acceptance of the *status quo* – even their own superiority or inferiority. Processes of socialisation, culture and ideology perpetuate exclusion and inequality by defining what is normal, acceptable and safe” [(25), p. 29]. Changing the form of power requires enabling citizens to envision a different future and ways to break away from disempowering social norms and to feel entitled to claim their rights as citizens. Different approaches to shifting forms of power include: advocacy, mobilizing and collective action, consciousness building to expose and change hidden power, and linking together various power shifting initiatives.

Given the increasing move to austerity politics in the Global North, it is timely to consider the detrimental impacts that power imbalances are having in both the North and South. As Amartya Sen explains, “As we would expect, the poor countries in Africa or Asia or Latin America provide crudely obvious illustrations of severe deprivation, but the phenomenon is present even in the richest countries. Indeed, the deprived groups in the ‘First World’ live, in many ways, in the ‘Third’” (26). Under increasing austerity measures in the North, for example, in Greece and the UK, health and social services for marginalized communities are reduced. In the UK, austerity is intensifying the pressures from rising food and fuel costs, increasing personal indebtedness, and declining government support to social security programs, including those for people seeking employment, seniors, and children (27). Furthermore, downloaded responsibilities from the national to local governments are, “… enabling both the state to retreat from responsibilities and food businesses to gain from improving corporate social responsibility (CSR)” [(27), p. 3623/6066].

At the same time, the already inadequate monies transferred from north to south are in decline. The power struggles between the need for basic sustenance by the marginalized and economic liberalization supported by the affluent converge to produce widening food insecurity and related health challenges. Together, this points to the need for structural change to realize a more just society, “… it seems obvious that tackling poverty and inequality is central to any good faith effort to protect the rights of the poor … It may well prove to be the biggest threat to recent gains in both health and human rights” [(1), p. 11]. Food security and children’s rights, internationally and in Canada, help us understand the tensions between market forces and human rights.

## The United Nations Convention on the Rights of the Child and Food Insecurity in Canada

As the most widely ratified international convention, the UN CRC provides a point of reference for the rights of children around the world and complements many of the provisions laid out earlier in the International Covenant on Economic, Social and Cultural Rights (ICESCR). Article 2 of the CRC sets out the foundational rights for each child to live without discrimination, “… irrespective of the child’s or his or her parent’s or legal guardian’s race, color, sex, language, religion, political or other opinion, national, ethnic or social origin, property, disability, birth or other status” (28). Article 24 is especially relevant as it frames the context for respecting the right to health and health care, as well as clean water and healthy food. It states that,

1. States Parties recognize the right of the child to the enjoyment of the highest attainable standard of health and to facilities for the treatment of illness and rehabilitation of health. States Parties shall strive to ensure that no child is deprived of his or her right of access to such health-care services.
2. States Parties shall pursue full implementation of this right and, in particular, shall take appropriate measures:
  (a) To diminish infant and child mortality; …
  (b) *To combat disease and malnutrition*, including … *through the provision of adequate nutritious foods and clean drinking water* …;
  (c) To ensure appropriate pre-natal and post-natal health care for mothers;
  (d) To ensure that all segments of society, in particular parents and children, are informed, have access to education, and are supported in the use of basic knowledge of *child health and nutrition*, the advantages of breastfeeding, hygiene, and environmental sanitation [(28), emphasis added to original].

The convention is clear that state governments have an obligation to provide families and children with the support needed to ensure that their nutrition is provided for as part of safeguarding good health. While recognizing the important work that has taken place through the robust global movement centered on the ICESCR since the 1990s and the UN-FAO Right to Food unit and the Voluntary Guidelines, this paper instead uses the CRC in Canada as an analytical frame to focus specifically on food security for children and related problems in achieving this human right as an alternative way into an exploration of rights and responsibilities.

In 2014, 25 years after the convention was ratified, UNICEF, the UN Children’s Emergency Fund reviewed the progress and challenges for child health and welfare for middle- and low-income countries. In its assessment, UNICEF reported that global progress had been achieved in some areas. Between 1989 and 2014, mortality rates for children under the age of 5 years were nearly halved, levels of antenatal care increased from 65 to 83%, and breast-feeding rates increased in LDCs from 38 to 50%. While recognizing these significant accomplishments, UNICEF also points out that more needs to be done. In particular, the importance of achieving food security cannot be overstated as malnutrition is directly or indirectly responsible for nearly half of all deaths in children under the age of 5 years, and in 2013, one quarter of all children were stunted [(29), p. 16].

While the UNICEF report did not comment on the status of “developed” countries including Canada, a UN panel of experts did review Canada’s compliance with the CRC, specifically whether children are food secure. The reviews were submitted to a federal government committed to free market, small government focused policies. Stephen Harper, the former Canadian Prime Minister who led the federal government during this time, was quoted as saying,

Human rights commissions, as they are evolving, are an attack on our fundamental freedoms and the basic existence of a democratic society … It is in fact totalitarianism. I find this is very scary stuff (30).

The following examples from Canada highlight the need for oversight and international reporting on all countries, not just low- and middle-income countries, by international bodies such as the UN. Given the call for increased austerity in some quarters in the Global North and declining levels of support for social programs (22, 27, 31), it is hoped that Canadian example can help focus attention on important power gaps that need to be filled, so that fundamental rights, including the right to food for children, cannot be pushed out of sight and ignored.

### Canada and the Convention on the Rights of the Child

Canada ratified the CRC December 13, 1991. Since then, some measures have been put into place to meet this commitment, including policies to enhance children’s rights with respect to military deployment to conflict zones, the provision of advocates for children in government care, and increasing the number of children who attend schools where their perspectives are respected. While these achievements are important, they are not sufficient to meet the commitments in the CRC. The lack of commitment is reflected in part by the high rates of food insecurity and the fact that, “… some children suffer from poverty, homelessness, abuse, neglect, preventable diseases and unequal access to good quality education, protection and justice systems” (32). A cornerstone of the previous federal government’s strategy for children was the “National Plan of Action for Children, a Canada Fit for Children” subtitled “Canada’s Plan of Action in Response to the May 2002 UN special session on children.” One pillar of this program was the “Universal Child Care Benefit” a tax credit based on the number of children and status of the family (married, living common law, widowed, divorced, separated, single). This benefit is accessed based on income, and parents are only eligible if they file a tax return.

Support varied and included both federal and provincial contributions (Table 1). While a universal child benefit is laudable – and has been in place in Canada on and off since the end of WWII, thus predating the CRC – the amounts reported in Table 1 were inadequate to ensure food security to Canadian children for families with no income (7, 34). And this is not an insignificant number of Canadians. In 2011, Statistics Canada reported that over 18% of people under the age of 6 years of age lived in households that fell into the “low income after-tax” category (35). In addition, the “Universal Child Care Credit” is not universally accessible. It requires parents submit an income tax form, and so, it discriminates based on access to technology and the capacity to complete the form, and assumes either English or French language skills.

**Table 1:** **Sample universal child care benefits (Canada Revenue Agency (33))**.

	Couple (married or common law), one child, no income	Single-parent family with a child with a disability
Federal contribution	$156.24	$573.10
Provincial contribution	$55.66	$111.30
Total monthly support	$211.90	$684.40

Two other pillars that addressed CRC commitments were childcare and early childhood education. The extent to which school nutrition programs are part of childcare can address food security, although the primary mandate related to CRC for childcare is to ensure affordability and consistency across the Canadian provinces and territories. While there are piecemeal programs in place in various communities, there is no universal, national school nutrition program of any kind in Canada that would be comparable to those available in the US, the UK, and some EU countries.

As a party to the CRC, Canada’s children’s rights performance is reviewed by an international body of experts from the UN who assess progress and compliance, as well as make recommendations about areas for improvement. The UN expert reviews are based on documents provided by the government and relevant organizations. The most recent review was submitted to Canada in 2012 and builds on two previous assessments; it reiterates areas where Canada can improve (36). That the Canadian federal government missed its third reporting deadline and rolled that into its fourth report could be said to reflect a lack of commitment to recognizing and giving importance to the rights of children.

In the context of rights, there are several key recommendations with respect to food security for children in this report. First, the UN review urges the federal government to pass legislation to entrench the rights spelled out in the CRC into Canadian law. This would provide a critical reference point for all levels of governments in considering any issue related to children’s rights in the future. The CRC review also recommends a national strategy on the Rights of the Child and suggests that this strategy should be consistent with the CRC to provide national congruence and compliance with the CRC. Furthermore, it recommends that the Canadian federal government meet its obligations to the CRC by establishing a national coordination body to liaise with the provinces and territories and provide adequate budgetary, data gathering, and reporting support. Given the vast Canadian expanse both in terms of geography and diversity, this is an important recommendation to ensure that the rights of all children are protected. The UN review committee also proposed that the federal government set up an independent national ombudsman to ensure progress with respect to the Convention. They also underscored the need for equal treatment of all children in Canada and point in particular to Aboriginal, minority groups, and children with disabilities as requiring additional support. With respect to all of these initiatives, the UN reviewers reiterated repeatedly throughout their assessment that,

… the State party *****allocate adequate human, technical and financial resources***** for the implementation, monitoring and evaluation of this comprehensive strategy and related provincial and territorial plans. [(36), pp. 2 and 3, emphasis added to original text]

The Canadian Coalition for the Rights of Children (CCRC), a not-for-profit advocacy organization, actively provided input into the consultation process. In response to and building on the first two reviews, the coalition authored a report titled “10 Steps for Children in Canada,” many of which reiterate or even extend the UN recommendations [Table 2; Ref. (37)]. Unfortunately, the federal government has failed to address most of the concerns raised by the UN review panel and the CCRC.

**Table 2:** **Ten steps for children in Canada (Table 2 is based on information derived from documents created by the Canadian Coalition for the Rights of Children (37))**.

1. Collect accurate data, analyze it, and publicly report on the situation of children
2. Create a consistent framework for policies that affect children
3. Implement a national strategy to prevent all forms of violence against children
4. Take immediate action on specific policy changes identified in the review
5. Establish a national ombudsperson for children
6. Ensure equitable treatment for indigenous children and other minority groups
7. Consider the best interests of the child and views of the child in all decisions
8. Inform children about their rights and train the adults who work with them
9. Provide access to affordable, quality childcare
10. Make the youth criminal justice system consistent with the convention

While all of these recommendations are critically important, the next section will focus on one dimension – that is, how the violation of these rights is reflected through food insecurity for too many Canadian children and their families.

#### Canada, the Right to Food and Food Insecurity

Food insecurity in Canada was made more visible as a result of the 2012 visit of Olivier de Schutter, then UN Special Rapporteur on the Right to Food. de Schutter was invited to make his visit by the Government of Canada and was hosted by the federal Department of Foreign Affairs and International Trade. During his visit, he traveled to five cities across the country, convened eight civil society meetings, and met with dozens of Aboriginal groups and communities in Quebec, Ontario, Manitoba, and Alberta. He spoke with officials from seven federal departments and multiple provincial ministries and municipal offices and received written submissions. As a result of this wide-ranging consultation, de Schutter was positioned to provide a very thorough and authoritative evaluation of food insecurity in Canada. He reported that,

A growing number of people across Canada remain unable to meet their basic food needs. In 2007/2008, 7.7 per cent of households reported experiencing moderate or severe food insecurity, approximately 1.92 million people, aged 12 or older, lived in food-insecure households and a staggering one in 10 families, with at least one child under the age of 6, were food insecure. Furthermore, in 2007/2008, 55 per cent of households in which the main source of income was social assistance were food insecure, the result of a pronounced discrepancy between social assistance levels and the rising costs of living [(34), p. 2].

de Schutter also pointed out the particular vulnerability of low-income households, women single-headed households, and Aboriginal communities with, for example, one in four single-parent households with children as food insecure (34). More recent numbers paint an even bleaker picture. In 2011–2012, 8.3% of households were moderately to severely food insecure, and 2.2 million people over 12 years of age were living with food insecurity (34). Additionally, food insecurity levels in the Canadian North can be as high as 69% in Aboriginal communities (38). Finally, in 2013, more than 840,000 Canadian households used food banks each month with one-third of these including children (39).

The government reaction to de Schutter’s assessment, as reported by the national broadcaster, was for then Immigration Minister, Jason Kenney, to say,

Canada sends billions of dollars of food aid to developing countries around the world where people are starving … It would be our hope that the contributions we make to the United Nations are used to help starving people in developing countries, not to give lectures to wealthy and developed countries like Canada. And I think this is a discredit to the United Nations. (40)

This article went on to explain,

… most of his [de Schutter’s] missions are in developing countries, but he estimates Canada has two to three million people who can’t afford the diets they need to lead healthy lives … “The right to food is about politics. It’s not about technicalities. It’s a matter of principle and it’s a matter of political will. I think these comments are symptomatic of the very problem that it is my duty to address” (40).

These comments illustrate the refusal by the Canadian federal government to address basic human rights, including a child’s right to food.

This is consistent with work by Riches, Silvasti, and others that documents the de-politicization of food security, the downloading of services, and the use of food banks and other charitable organizations to fill the gap federal governments are obliged to meet to ensure food security as a right. Similar to pressures on social support mechanisms in other jurisdictions [e.g., Ref. (20)], charitable groups, such as food banks, have become dumping grounds for corporate food surpluses as food banking is cheaper and more socially palatable than sending unsalable food to landfill (7).^1^ This adds to the ill-informed, neoliberal supported impression that food charity organizations are “common sense” approaches that provide adequate aid to “needy” clients [(7), p. 1307/6066], so that “… corporate food charity both masks the ineffectiveness of food banking and obstructs the development of public policies directed at the achievement of food security and social justice for all … [and] further entrench an unaccountable charitable food banking system” [(7), p. 1428/6066]. Furthermore, the poor quality of this food and unreliable access means people who depend on these organizations do not have the food they need to meet their physical and mental health needs (7). This approach reflects the power conflict between rights and neoliberal politics as citizenship and rights are willfully entangled with consumption and economics,

At present the right to food is displaced by a private business relationship created through the purchasing power of money in the food market. When citizens are made to be consumers, rights are easily made to be business transactions or contractual agreements (41). The right to choose and human dignity is lost [(41), p. 563/6066]

Given Canada’s status as an OECD country, it might be assumed that its citizens do not face food insecurity challenges. In this context, the Canadian example of over a decade of neoliberal, small government that disempowered its most vulnerable citizens serves as warning for other countries about the impacts of ignoring the rights of its citizens in general, but in particular its children. This is especially concerning given the growing emphasis in OECD countries on small government and the links to EU austerity programs (27).

## Integrating Multiple Levels to Exercise New Forms of Power

This section reviews alternative food initiatives in Canada that have moved into the vacuum created by State neglect of human rights. These community-based initiatives provide good, healthy food, and also educate citizens about food and advocate for change both provincially and federally. Through collective, multileveled power leveraging, these examples offer possible ways forward in meeting human rights obligations through food.

These examples come from an analysis of online sources, academic, and gray literatures. This is combined with knowledge from writing about policy and regulatory contexts in Canada in both academic papers and reports (42). The analysis adds to firsthand knowledge of the not-for-profit, FoodShare where there has been an on-going research partnership for more than 5 years; participation in a meeting with UN Special Rapporteur to the Right to Food, Olivier de Schutter, as part of his 2012 visit to Canada; contributions to Food Secure Canada’s People’s Food Policy process and on-going support of their organization through student placements; and work as a supportive observer to the Civil Society Mechanism of the UN-World Committee on Food Security. The case study in Windsor was part of a larger project in our research group that explored innovative governance opportunities in the Ontario, Canada context.

### Respecting and Fostering the Right to Food in Canada: Actions at the Community Level

Consistent with the emergence of CSO initiatives in other countries (20, 43–46), the lack of attention to food security by the Canadian federal government coupled with the disempowering impacts of food charity organizations (7), has resulted in many community-based organizations in Canada stepping in to fill the void. This section presents two examples of how communities and community organizations on the ground are trying to ensure food security in ways that empower communities so they can be healthier and more self-determining. The first example is FoodShare, an internationally recognized leader in community-scale food initiatives located in Toronto and their Good Food program. The second example is the Windsor School Food Nutrition Program, an innovative pilot project that provides affordable, healthy snacks to school children, as well as vocational training to high school students. A third example discusses a federal government-sponsored program, the Canada Prenatal Nutrition Program. While this initiative is no longer funded, it offers insights into the potential for well-developed federal programs to foster improved health and well-being for mothers and their babies and to realize their rights to food and health. All of these programs exercise power at multiple levels to address pressing social concerns and create space for these issues in public discourse.

#### Multilevel Empowerment: Bridging the Power Gap

While FoodShare was mandated in 1985 to address food aid and emergency food delivery, within a year it shifted its focus and,

… began its advocacy work working towards long-term solutions to hunger and statement of objectives included “lobbying for income distribution, housing, social assistance and minimum wage rates, day care, and work assistance programs”. Donna MacDonald, a community activist, was hired as FoodShare’s first Executive Director, bringing years of experience working on housing, health and income security issues. Under her leadership from 1985 to 1988, FoodShare generated an understanding of hunger and poverty issues and developed and implemented programs and solutions in partnership with communities using a grassroots community development approach. In 1987 Executive Director Donna MacDonald proposed a funding structure splitting operating costs for FoodShare ($89,000) between Metro Toronto 30% and the City of Toronto 35%. The remainder (35%) would be fundraised by the organization (47).

FoodShare has grown since the mid-1980s from its initial focus on food relief to an organization that created the first Food Box program, a subsidized fresh produce box in 1992. To supply the Good Food Box, FoodShare developed direct sourcing relationships with local farmers and wholesale distributors at the Ontario Food Terminal and became a food distribution hub and community resource/facilitator for programs ranging from mobile markets, providing food education, and both initiating and supporting school nutrition programs. In 2014, it had revenues of $6.46 million Cdn. The primary funding sources were 40% program sales, 32% foundations (including United Way), 11% individuals, 7% capital assets, 5% municipal with the remaining 5% coming from federal and provincial funding, events, interest, and bequests. On the expenditure side, funding was allocated as 46% program staffing, 32% program costs, 5% warehouse and transportation costs, with the remaining 17% split between amortization, fundraising and communications, volunteers and office, and administration. What these numbers tell us is that FoodShare generates nearly half its revenue from its own sales, and that its other funding comes from a variety of sources making it more flexible and resilient. FoodShare is motivated by food justice as an approach that,

… creates and supports local solutions to address systems of oppression and exclusion in the food system … [with] a priority to work with communities of color, new Canadians, indigenous communities and other equity seeking groups by providing organizational resources to support community-led food access groups … Food justice is at the core of FoodShare’s work and mandate. We facilitate empowerment and community development from the ground up, striving to achieve equitable access to good healthy food for traditionally underserved communities. We are working to build a more democratic, just and equitable Ontario, through trainings, workshops and community animation (48).

FoodShare offers a Good Food Program that includes four different community programs: the Good Food Markets, the Good Food Box, the Good Food bulk program, and Good Food Mobile Markets. Together, these programs cover the cost of the more than 2 million pounds of produce valued at more the $1.6 million delivered to more than 288 schools and non-profit agencies, 22 mobile and Good Food Markets, and more the 85,000 Good Food boxes, in 2014. The staff and volunteer time needed to run these programs depends on foundations, government programs, and goodwill. One-third of the dollar value of the food purchased was from local farmers who were paid a fair price for their produce (49). The Good Food Box is available to anyone in the Toronto area for a set price regardless of income. At the time of writing, boxes cost between $13 and $34 and are filled using fresh fruits and vegetables from local farmers and the Ontario Food Terminal. Produce is purchased according to the following priorities, “quality, value, culturally appropriate food, local and seasonal, sustainable growing practices, reduced packaging, and fair trade” (48).

FoodShare is active on multiple fronts and works to enable community conversations around food rights. For example, it hosted Olivier de Schutter in 2012 and provided input into the consultation on the state of food security in Canada. It was also involved in the development of the provincial Local Food Act, the first of its kind in North America, and has been instrumental in the creation of the model for student nutrition programs (SNP), which influenced the creation of a province-wide school nutrition program. Student Nutrition Toronto (SNT), a partnership between Toronto Public Health, Toronto District School Board, Toronto Catholic District School Board, Toronto Foundation for Student Success, The Angel Foundation for Learning, and FoodShare supports 700 programs across Toronto which collectively serve nearly 149,000 healthy, nutritious meals every school day to children and youth. FoodShare works with schools toward long-term viability of each program (50). As a lead for the SNT Community Development Team, FoodShare provides support through its community development animators to individual school and community-based student nutrition sites. As well, staff at FoodShare:

… continue to advocate for funding and infrastructure for community programs, and for a federal universal child nutrition program, which would ensure that every child and youth receives a nutritious meal every day that they are in school. (51)

An even more comprehensive program that builds on existing knowledge and infrastructure for healthy food delivery are FoodShare’s Good Food Cafés. This pilot initiative emerged in the wake of provincial regulations that required school meal providers to avoid serving high fat, salt, and sugary foods and foods of low nutritional value to primary and high school students (52). This made on-site cafeterias unprofitable for many school food service providers, as they relied on ready-made or cheap foods, including French fries and sugary drinks to generate a profit. As a result, FoodShare was invited in to fill the gap in first one, and now four, schools. The Good Food Cafés are committed to,

“… universal and healthy school cafeteria, serving attractive, delicious and nutritious food that students choose to eat and that is simple to prepare, proving that … good for you can be easy for schools to prepare, and tasty too … Students enjoy a diverse menu … Hot meals are accompanied by salads, fresh vegetables, rice, grains, beans and fresh fruit.’’ The Good Food Café serves over 250 students each school day in two public schools (51).

The SNP that FoodShare supports in Toronto are part of a province-wide initiative funded by the Government of Ontario through its Ministry of Children and Youth Services. Fourteen regional agencies across the province administer $8.5 million in annual grant allocations in collaboration with community partners, including officials from public health, school boards, as well as other private and public sector actors (53). As reported in a case study by Nelson in Windsor Ontario, the Victoria Order of Nurses (VON) is the lead agency (54). The pilot project has proven to be innovative for a number of reasons. This initiative addressed several challenges that emerge for schools, as they do their best to deliver nutrition programs by centralizing purchasing, reducing the need for volunteers, purchasing local produce as much as possible, providing training opportunities for youth, and developing relationships between stakeholders (54). Centralized procurement helped reduce costs and allowed the food to be stored at the Unemployment Help Centre (UHC), where youth from the Specialist High Skills Major Co-op program prepare all of the snacks, giving them hands-on experience in food service. In addition, the students earn four high school credits, a college credit, and other professional certifications. As the program evolved, they were also engaged to prepare hot meals for seniors, a mobile food bank, and community events. Preparing meals allowed this innovative program that started with school snacks to create a social enterprise. Now, in addition to the snacks for six schools, an average of 200 meals per day is also prepared. Given a $3/meal saving through using the UHC prepared food, based on the pilot program, the VON is able to feed more children healthy food in schools, purchase more produce from local farmers, provide hands-on experience to youth so they can develop food services skills and be more employable, and also build a stronger sense of community. In addition, it is estimated that the program could grow to 1000 meals per day. Given the existing infrastructure at the UHC kitchen, food processing could also be scaled up, offering another revenue stream if the processed food products were sold. Importantly, the food is all prepared from scratch using fresh ingredients, including breads, soup stocks, and pickles, so that no processed food is used (54).

Over the years, there have been a number of programs to support various aspects of child nutrition at the federal level that no longer exist (41). One example is the Prenatal Nutrition Program (PNP). The summative evaluation assessment for this program reported that most people participating in the program from 2002 to 2006 were from at-risk populations: nearly 28% were recent immigrants (fewer than 10 years in Canada), close to 25% were Aboriginal, over 80% of participants had household incomes of $1,900 or less per month, nearly 10% reported no income, nearly 12% were teenagers, and over 40% had consumed alcohol since becoming pregnant, with half reporting having at least five drinks in 1 day. This program was making an important contribution to maternal and infant health. Participants with more time in the program were more likely to have made positive changes and have healthier babies. These women had increased vitamin or mineral supplements use, decreased smoking, stopped consuming alcohol, were 26% less likely to give birth preterm, 34% less likely to have a low birth weight baby, and 11% less likely to have a baby born small for his or her gestational age. They also had higher breast-feeding initiation and breastfed their babies longer. Empowering women through the PNP program allowed them to be more active participants in assuring health for themselves and their babies (55). This would also have contributed to enhanced food security for at-risk babies and their mothers.

These three examples point to how organizations and programs can help redress human rights violations through the provision of, for example, food to children and families. This can be an important balancing force in the realization of rights as organizations and their networks become agents of change. Additionally, organizations that balance their resources between public sources, philanthropic, and individual donations, as well as money earned from their own programs, may offer more resilient long-term answers for food system viability and the carving out of more secure, powerful spaces. This could also enable their recognition as “catalysts for positive change” and being “valued” according to the multifunctional benefits they provide (56). However, to ensure states meet their obligations, structural change is needed. This is addressed next.

### Exercising New Power

From 2006 to 2015, the Canadian federal government under Prime Minister Harper increasingly adopted a pervasive small government, austerity approach. In this paper, we have discussed how this was apparent through high food insecurity rates and more generally through a lack of attention to the rights of children. The ideological perspective informing the government’s discourse at that time is captured in a Huffington Post article reporting on Canada’s role in the international community and the EU fiscal crisis,

Even with youth unemployment rates in a number of countries at 50% or higher, Ottawa [Canada’s national capital] has repeatedly supported the German-led push for other European governments to cut social spending. The Conservatives [governing State party] have backed this thinly veiled ruling class effort to weaken labor’s bargaining position and roll back the European welfare state …. During a June 2011 visit to Athens, Harper [Canadian Prime Minister] forcefully backed austerity measures.

While supporting austerity measures, the Conservatives have publicly opposed efforts to tax and regulate the banks largely responsible for the economic collapse … The Harper government has consistently supported Canada’s banks and global investor class. In fact, their entire foreign policy is largely designed around the question: how can we make the world’s richest 0.1% even richer? (31).

Applying a power cube lens to understand the Canadian government’s approach to food security, we can see that the government used both visible and hidden power to privilege discourse around free markets and downplay the need for attention to social justice issues. Decision-making was enclosed at the federal level, as it denied funding to programs that supported socially just initiatives such as food security and prenatal nutrition and privileged solutions such as food banks. This meant a blinkered approach to social welfare and is problematic in ensuring human rights. While it is the obligation of the signatory States to the CRC to ensure these rights for children, when this duty is ignored by the State, CSOs can both step in to ensure needs are met and move the political needle in a more accountable direction. FoodShare, the SNP, and the PNP all demonstrate that new spaces can be established to both activate for and provide for enhanced food security for children. Using power across multiple scales allowed for the improvement of access to more healthy food for more children and opened up spaces for access to local healthy food at the provincial scale.

#### Food Secure Canada and a National Food Strategy

Food Secure Canada was founded in 2003. In 2008, it engaged in a country-wide consultation that resulted in the People’s Food Policy (12). More recently, Food Secure Canada has been able to leverage its broad community and civil society base to raise food security as an election issue at the municipal, provincial, and national scales. A particularly notable achievement was that, during the 2015 federal election campaign, three of the four national parties included versions of a national food policy in their election platforms. During the lead-up to that election, Food Secure Canada led its own campaign to raise the profile of food security and food-related matters called “Eat Think Vote”. The initiative logged 68 events that included the direct participation of 164 candidates and 4,461 others, reaching more than a million Canadians through radio, television, newspapers, and social media (57).

Building upon their successes developing the PFP and engaging in election-period advocacy, FSC continues its work on a national food framework. Their initiative draws on the work of McNicoll (58) and is centered on a call for the creation of a National Food Strategy (NFS), an initiative called for as part of the FSC PFP recommendations. While a National Ministry of Food may be a long term goal for Food Secure Canada, energy is currently directed towards advocating for a NFS. The merits of a NFS for Canada would be the ability to address complex food system issues in a connected, multi-scaled, comprehensive way that would foster the “dialog and collective decision-making” called for by FSC and others. To do this effectively, ‘‘a National Food Policy Council’’ (NFPC) or something similar, would need to be created and include diverse experts, cross policy domains, and receive long-term core funding to position it to foster long-term proactive policy development (57). As summed up in a recent document assessing the need for the NFPC in Canada,

It brings together diverse experts from across the food system to find solutions that take a variety of perspectives into account; it engages Canadians in food policy issues and democratises our food system; and it leads the way toward a healthier, more sustainable, and more economically viable Canadian food system. It is time the Canadian government invest in a more sane and comprehensive approach to food policy: establishing a national FPC as an independent government institution informs and advises policymakers and elected officials on sound food policy decisions and holds them accountable. An arms-length organisation reporting to Parliament and to all ministries responsible for food policy-making through the Ministry of Health is the most effective option: it is a model that has proven by other organisations; would receive stable funding and staff; have a strong network and grassroots support; and, as an independent body, allows for comprehensive food-systems thinking [(57), p. 11].

Thus, FSC offers a way to unite multiple actors from various sectors focused on SFS transformation through political activism at multiple scales. This coherence provides a collective voice and keeps food security issues on the political agenda. This initiative, if successful, would help ensure the right to food for children. FSC is remarkable for its collaborative, inclusive, flexible approach in the context of a thorny national challenge. The People Food Policy is notable as it offers a model for the creation of a collective approach to the CRC and more specifically food security across the country. This initiative is an excellent example of Gaventa’s multiple facets of power that can be leveraged for transformation. By occupying power spaces in a new way, FSC has been able to identify shared priorities and build collaborative opportunities for future change. This demonstrates the potential for considering the multi-scaled points of entry that can enable power shifts so that, by negotiating in an open and transparent way, new discourses can be established.

## Discussion and Conclusion

Transformative, fundamental change happens, I suggest, in those rare moments when social movements or social actors are able to work effectively across each of the dimensions simultaneously, i.e. when they are able to link the demands for opening previously closed spaces with people’s action in their own spaces; to span across local and global action, and to challenge visible, hidden and invisible power simultaneously … successful change is about getting each of the pieces on each dimension of the cube to align with each other, simultaneously [(23), p. 30].

The complex nature of power means that structural transformation is not easy or frequent. It requires the convergence and alignment of appropriate spaces, levels, and forms of power to bring about change. Based on the “Power cube,” we see that to enact power and bring about transformation, individuals/institutions need to be able to coenact all forms of power. They would participate in hidden power so they are able to contribute to the political agenda. Through visible power, they would support the decision-making that results in laws, regulations, and ultimately structures. And, they would use invisible power to shape beliefs, norms, and ideologies that guide public discourse. They would access spaces of power so that claimed/created spaces exist across multiple levels and ensure the forms of power are transparent and legitimate.

It is valuable to reflect on the real need for additional supports for ratified international agreements and recall Narula’s words cited at the beginning of this paper that refer to the difficulty, “wherein top-down processes can effectively navigate entrenched power dynamics (18) … the State and its ruling elite are not neutral agents of social change.” To the contrary, State actors and domestic elites often stand to benefit from rights-violating policies and practices [see also Ref. (7, 19)]. As we have seen, situating too much power in the hands of the State limits the capacity of individuals and grassroots organizations to insert themselves and effect transformation. Given moves in the EU and the UK toward austerity measures and the US refusal to sign on to and/or ratify many international conventions including the International Criminal Court and the CRC, this power gap needs to be addressed. The Canadian example points to the need for multiple checks and balances throughout the spaces, levels, and forms of power so that human rights and adopted international conventions cannot be ignored or violated. Non-government actors need to have the space to act legitimately to support change and provide oversight of government (in)action. The power cube makes it clear that CSOs need to have the capacity to claim power and inhabit spaces across multiple scales so they can monitor and support human rights, help shape public rights-based discourse, and ensure that the right to food for children is a social expectation and not negotiable.

The Canadian example of food security as one facet of children’s rights raises questions about what happens when powerful actors prevent the realization of human rights. The powerful actor, in this case the federal government, is the entity responsible to ensure these rights are respected. Given the State’s failure, the example of the CRC and food security is instructive for two of reasons. First, it provides additional insights into some of the power dynamic process when two epistemologies clash. Second, it presents pathways for the realization of more robust mechanisms that could support human rights.

First, are the dueling epistemological tensions between the then federally supported neoliberal, free market government and the community, provincial, and international actors aligned around social justice, human rights, and rights-based approaches. As we have seen, the federal government created closed spaces where hidden forms of power were used to obscure decisions about laws and programs. For example, reports to the UN were delayed and constructive criticism from internationally respected sources was ignored. At the same time, there was a steady increase in government constructed public discourse (a form of invisible power) in support of neoliberal, atomistic approaches that valued and, in the end, privileged free market principles over the protection of human rights. The neoliberal discourse allowed for the general acceptance of food banks as long-term solutions to food insecurity and the public denunciation of de Schutter’s report. Further, the government used its hidden power to gradually change the laws, policies, and programs so that after 9 years, structures that should have protected, respected, and fulfilled human rights were impacted. The cancelation of the PNP and restricted access for families to the Universal Child Care Benefit are examples of narrowed resources in support of family well-being. In contrast, there was support from community-based CSOs and other levels of government, including the UN review, for interventions that enacted food security. Existing and emerging pilot projects in Ontario around school food programs, while not large-scale enough to reverse the trend toward increasing food insecurity, do challenge the existing system and point to the potential to support human rights. The School Nutrition Program initiatives, FoodShare Good Food Programs and the Canadian Prenatal Nutrition Program are examples of initiatives that offer promise for enhancing nutrition for all children. This can go some way to improving food security for the most vulnerable Canadians. And, while these programs are not universal, the Ontario Local Food Act and the School Food Program Guidelines as advocated for by FoodShare and other CSOs, and FSC initiatives around the creation of a NFS that emerged from grassroots consultation, and subsequent engagement of the public and politicians during recent elections have all raised the profile of local food and the right to food. Provincially, there are shifts to support the public procurement of local food as healthy choices for school food programs and other public institutions. FoodShare offers a model for financial diversification, both in terms of program funding and delivery. Collectively, these programs provide examples of how to move forward in dealing with the multiple challenges that have emerged in Canada through more open, inclusive collaboration across multiple power levels that can ultimately translate into structural change.

Ideally, as presented earlier, several pieces need to be in place for this power shift to be secured. As contained in the UN, CCRC, and FSC recommendations, children’s rights need to be entrenched in federal law as a rights-based reference point. A national strategy on the rights of the child that links all levels of government with adequate financial, data and monitoring resources, and an ombudsman is required to oversee the process. Furthermore, these institutions could be reinforced through two mechanisms: joined-up policy and networked governance. MacRae et al. (22, 58, 60) describe the need for “joined-up policy” that, “… requires integration across jurisdictions, such as health, agriculture, environment and social policy, and can offer more sustainable and equitable food policy options” (p. 570). As suggested in the UN, FSC, and CCRC documents, integration across departments would embed these principles in appropriate government departments and would help to ensure a child-based rights lens was applied more comprehensively and consistently across multiple facets of society. Joined-up policy could go hand in hand with monitoring and justiciable recourse [(7), p. 613/6066, see also Ref. (59, 60)] but for people to obtain these rights, they need to be able to create and exercise their power. This is consistent with rights-based approaches that demand participation by and inclusion of vulnerable people whose rights have been violated or abused (61). Networked governance that puts power in the hands of people and CSOs may also be part of the solution. “In the last decade networks and networked actors have produced a new form of flexible, just-in-time political agency” [(21), p. 33]. As demonstrated by the multi-level challenges to policy and program from FoodShare, the SNP, and Food Secure Canada, these networks can provide a rights-based counterweight to neoliberal or other forces that work to deny citizens of their rights. According to a recent International Institute for Sustainable Development report, to be effective, networked governance actors need to create a collaborative, shared vision and build social capital so they can tackle complex problems through collective, strategic action (12). Notably, the example of scaling up of School Nutrition Programs to networked, provincial programs points to a gathering momentum and a more active form of power that could be transformative. Different approaches to shifting forms of power include advocacy, mobilizing and collective action, consciousness building to expose, and change as mechanisms that weave together of various power shifting initiatives. The creation of political momentum by FSC is another good example of this collaborative, collective power. Well-resourced networks of NGOs can take action to “pave the way” for State action by building trust, showing good practice, and demonstrating effective ways to achieve human rights. NGOs can also help people understand through education, that they can hold and claim their rights. These functions are in addition to any lobbying that the NGO does for directly respecting, protecting, and fulfilling human rights in the political sphere,

The tie-up between grassroots campaigning on food poverty, and those working for more ethically, sustainably produced food at affordable prices is beginning to emerge, but the issue represents a key component of realizing the right to food: that people be enabled to engage and feed themselves in ways they see fit, and so as to achieve wellbeing and potential for themselves and the planet [(62), p. 3670/6066].

FoodShare and the Windsor School Nutrition project demonstrate both the need and opportunity to leverage the multifunctional capacity of food to ensure human rights, including food security, human health, environmental well-being, political engagement, and community economic justice. Further, the realization of rights could be supported by provinces and through municipal initiatives such as food policy councils; the more points of support, the less likely it would be dismantled, an under-recognized benefit of joined-up policy (59, 60). As Dowler suggests,

The emergence of ‘hybrid’ initiatives, which engage networked members in policy analysis and advocacy as well as practical, ground level response, has potential to offer voice, creative ideas and shared possibilities for action [(62), p. 3651/6066].

Caution is needed though as prominent networks also become targets for competing interests (62, 63).

Recalling earlier discussion about the global effects of the industrial food system and concentration of power in the hands of corporations, the intertwining of neoliberal government and large scale agri-food, it is again clear how corporations assert their interests through pressure on governments to comply with practices that support their agenda and violate principles of social justice and human rights. Until this is addressed, the world is unlikely to make the progress it needs on issues related to health, justice, and the environment. The Global North needs to uphold the conventions that address poverty and limit the power of corporations so it is possible to respect human rights and avoid traversing irreversible ecological tipping points (6, 7, 64). This requires both sophisticated systems thinking across all forms, spaces, and levels of power in combination with determination and commitment to basic human rights. There are several international initiatives to address food insecurity in recent decades that offer interesting examples in this direction. Food activist initiatives including the Via Campesina and related food sovereignty movements push back against this blindness to social justice and human rights. There are also more specific institutional initiatives, since the 1990s, including the elaboration of key elements about the right to food in the ICESCR; the establishment in 2000 by the UN-Commission on Human Rights’ of the *Special Rapporteur on the Right to Food*; the creation of the UN-FAO Right to Food Unit and the adoption by member states of the Voluntary Guidelines on the Right to Food; a complaint and inquiry process for individuals through the UN General Assembly in cases where a person’s right to food has been violated [(7), p. 582/6066]; and the reform of the UN-World Committee on Food Security to include an active role for civil society (20). Importantly, the Voluntary Guidelines reinforce a state’s responsibility to, “respect, promote and protect … [and] are encouraged to apply a multi-stakeholder approach to national food security” [(7), p. 592/6066].

While Gaventa’s power cube makes the problems clearer and points to the gaps in power sharing, it also helps us find solutions to these challenges. Addressing inequality for marginalized communities requires that people have the power to ensure their basic rights. In the same way that Keynes advocated for policies that would smooth economic boom and bust cycles and provide more stability for (un)employment and social well-being as the world emerged from WWII, there needs to be attention to establishing the multi-faceted supports needed to ensure human rights, including food security, across all scales of government and civil society. Given the recently elected Canadian government priorities, perhaps there will be the required changes in this direction in Canada and the facets of the power cube will align to bring about transformation. The Mandate Letter to the Minister of Agriculture and Agri-Food instructs him to, “[d]evelop a food policy that promotes healthy living and safe food” (65), while the Mandate Letter to the Minister of Families, Children, and Social Development states, “[a]ll Canadian children deserve a real and fair chance to succeed, and all Canadians should be able to live with dignity” (66). Bringing these two mandates into alignment through more joined-up policy could offer hope that universal food security for children will be achieved and that their rights will be respected. As well, there may now be an appetite for entrenching the right to food in the Canadian constitution (37, 41). Acting on guarantees of the rights of children is a necessary, foundational step in this direction. At the same time, fostering networks between robust CSOs at all scales through networked governance approaches could establish counterbalances for future governments less attuned to human rights obligations. That said, given the shifting terrain for federal governments it is critical to ensure power resides at multiple levels, so that human rights obligations are consistently realized and do not ebb and flow with changes in governments. Returning to the quotation from Paul Farmer at the beginning of this paper, instead of averting our gaze we need to focus it on who has the power and ensure it is being used for the betterment of all.

## Author Contributions

The author wrote the submitted paper on her own. While the author is grateful to Dr. Molly Anderson, Ms. Field and others at FoodShare for their comments, all errors and omissions are her own responsibility.

## Conflict of Interest Statement

The author declares that the research was conducted in the absence of any commercial or financial relationships that could be construed as a potential conflict of interest.

## Footnotes

^1^In 2012, 87% of food from Food Banks Canada came from the corporate sector [FBC 2012, 10 in Ref. (41)].
